# An investigation of genotype-phenotype association in a festulolium forage grass population containing genome-spanning *Festuca pratensis* chromosome segments in a *Lolium perenne* background

**DOI:** 10.1371/journal.pone.0207412

**Published:** 2018-11-14

**Authors:** John Harper, Dagmara Gasior, Ros Mathews, Ann Thomas, Caron Evans, Julie King, Ian King, Mike Humphreys, Ian Armstead

**Affiliations:** 1 Institute of Biological, Environmental and Rural Sciences, Aberystwyth University, Aberystwyth, United Kingdom; 2 School of Biosciences, University of Nottingham, Sutton Bonington, United Kingdom; Ankara University, TURKEY

## Abstract

Alien chromosome introgression is used for the transfer of beneficial traits in plant breeding. For temperate forage grasses, much of the work in this context has focused on species within the ryegrasses (*Lolium* spp.) and the closely related fescues (*Festuca* spp.) particularly with a view to combining high forage quality with reliability and enhanced environmental services. We have analysed a *L*. *perenne* (perennial ryegrass) population containing the majority of a *F*. *pratensis* (meadow fescue) genome as introgressed chromosome segments to identify a) marker-trait associations for nutrient use and abiotic stress response across the family, and b) to assess the effects of introgression of *F*. *pratensis* genomic regions on phenotype. Using container-based assays and a system of flowing solution culture, we looked at phenotype responses, including root growth, to nitrogen and phosphorus status in the growing medium and abiotic stresses within this festulolium family. A number of significant marker/trait associations were identified across the family for root biomass on chromosomes 2, 3 and 5 and for heading date on chromosome 2. Of particular interest was a region on chromosome 2 associated with increased root biomass in phosphorus-limited conditions derived from one of the *L*. *perenne* parents. A genotype containing *F*. *pratensis* chromosome 4 as a monosomic introgression showed increased tiller number, shoot and root growth and genotypes with *F*. *pratensis* chromosome segment introgressions at different ends of chromosome 4 exhibited differential phenotypes across a variety of test conditions. There was also a general negative correlation between the extent of the *F*. *pratensis* genome that had been introgressed and root-related trait performances. We conclude that 1) the identification of alleles affecting root growth has potential application in forage grass breeding and, 2) *F*. *pratensis* introgressions can enhance quantitative traits, however, introgression can also have more general negative effects.

## Introduction

Within Northern Europe and temperate areas worldwide, ryegrasses, particularly *Lolium perenne* (perennial ryegrass), are the most important forages in grassland agriculture and they are the target of sustained and competitive commercial breeding and seed production activities. The popularity of *L*. *perenne* in the farming sector is due to its combined qualities of forming a reliable perennial pasture, its responsiveness to fertilizer inputs in terms of biomass and quality, and the generation of good livestock performance in the form of enhanced meat and milk production. In more northerly and continental climates, however, the reliability of *L*. *perenne* declines and *Festuca pratensis* (meadow fescue), while lacking some of the quality characteristics of *L*. *perenne*, is often used as the key grass component of the sward due to its greater tolerance to low-temperature and related abiotic stresses. Similarly, in more southerly climates ryegrasses can exhibit sub-optimal performance in the face of heat and water stresses and polyploid fescues, such as *F*. *arundinacea* (tall fescue), may be preferred. As a consequence, it has been an aim of forage breeders for a number of years to exploit the inter-fertility of ryegrass and fescue species to combine their complementary and favourable attributes in ‘festulolium’ varieties, either using an amphiploid or an introgression breeding approach [1–6]. Additionally, with increasing climate uncertainty and the need to reduce inputs, the production of novel forage grasses, including festuloliums, which can perform well under these developing economic and environmental conditions is of fundamental importance. New and more responsive grass varieties will be key components in approaches to the development of more sustainable grasslands which can address the needs of grassland agriculture whilst also providing key ecosystems services, such as water and nutrient-use efficiency, hydrological management and soil stabilization [2,7–10].

In addition to their agronomic complementarities, ryegrasses and fescues in general, and *L*. *perenne* and *F*. *pratensis* in particular, also have karyotypic characteristics which have made them very useful tools in both molecular cytogenetics and comparative genomics of the grasses. These characteristics are that they combine a high degree of interfertility and interspecific chromosome recombination at meiosis with the ability of the analyst or breeder to distinguish the species-of-origin of chromosomes and chromosome segments in hybrids and their progeny using genomic *in situ* hybridisation (G*IS*H). This has been exploited in analyses of interspecific recombination [11–16], trait mapping [3,5,17–20] and in comparative genomics and linked candidate gene identification [21–23]. Ryegrasses and fescues are members of the Poodieae sub-family and, so, closely related to both the Triticeae cereals, the monocot model species *Brachypodium distachyon* and, more distantly, to rice. Diploid *L*. *perenne* and *F*. *pratensis* are also both intermediate genome size species (c. 2.5Gb; [24]) and represent interesting comparative ‘bridging’ species for Pooidieae genomics. In this context of comparative grass genomics, Harper et al. [25] reported the development of a monosomic introgression series for all 7 chromosomes of *F*. *pratensis* in the *L*. *perenne* background (2n = 2x = 13 *L*. *perenne* + 1 *F*. *pratensis* chromosomes). Subsequently, King et al. [22] reported the mapping of >1500 rice genome-anchored markers in progenies derived from each of these monosomic introgression genotypes. Each member of these derived families contained a single, or occasionally two, haploid, introgressed sub-chromosome segment(s) of *F*. *pratensis*. Across the whole of this population this covered the great majority of the complete *F*. *pratensis* genome. That report, along with the *L*. *perenne* GenomeZipper [26], established the comparative resources that have contributed to the development of the recently published anchored genome assemblies for *L*. *perenne* [24,27].

Hydroponic cultivation, using flowing solution culture (FSC), has been used in the evaluation of ryegrasses and other forage species for many years (*e*.*g*., [28–40]) often focussing on responses to nitrogen (N) and phosphorus (P) concentrations and forage grass/clover interactions. More recently as part of the plant breeding work at Aberystwyth, FSC has been used in a number of pre-breeding assessments of forage grass and legume germplasm, particularly in relation to identifying genotypes with the potential for improved N- and P-use efficiency. Thus, for perennial ryegrass, FSC has been established as a useful tool for discriminating differential responses to nutrient concentrations.

In the present study we have conducted a series of phenotype assays on this genetically and genomically characterised festulolium family to identify genomic regions associated with differential phenotypic responses to nutrient status in the growing medium and to abiotic stresses and to determine the degree to which the architecture of trait inheritance might be influenced by the presence of alien chromosomes or chromosome segments. The outcomes are discussed in terms of the potential and possible implications for forage grass breeding.

## Materials and methods

### Population development

The development of this mapping population has been described previously in detail [22,25]. The crossing programme can be summarized as follows and is illustrated in S1A Fig. A tetraploid *L*. *perenne* genotype from the variety Meltra (designated LpM) was crossed with a diploid *F*. *pratensis* genotype (designated Fp; derived from a seed accession of a Russian landrace, ABY-Bf1183) to derive a triploid genotype containing 2 haploid genomes of LpM and one of Fp. This triploid genotype was crossed with a diploid *L*. *perenne* from the variety Liprior (designated LpL) and 6 monosomic substitution genotypes (S1-S6) were identified within the progeny through molecular cytogenetic and molecular marker screening. Each of S1-S6 contained a different chromosome from the Fp haploid set (2n = 2x = 13 *L*. *perenne* + 1 *F*. *pratensis*). For S7, the first genotype identified as containing a complete introgressed Fp chromosome 7 (C7) also contained another small Fp-derived terminal segment from a different chromosome. This latter segment was removed while retaining the complete monosomic C7 introgression following a second round of back-crossing to LpL.

Each of S1-S7 were then further back-crossed to LpL, and *L*. *perenne* /*F*. *pratensis* recombinant genotypes from the whole Fp chromosome substitutions in S1-S7 were identified through further rounds of molecular cytogenetic and molecular marker screening (the progenies derived from the S1-S7 x LpL backcrosses were designated S1fam1-S7fam, respectively). The entire Fp genome was present across S1fam1-S7fam with the exception of the central section of chromosome 6 (C6) (S1B Fig). A further population with no *F*. *pratensis* introgression was developed for this study as follows. LpM was crossed with LpL to derive a triploid LpM/LpM/LpL hybrid. This was then backcrossed onto LpL and the resulting genotypes designated as the LpCon family. The numbers of genotypes derived from each of S1-S7 (*i*.*e*., S1fam-S7fam) and Lpcon were 8, 13, 23, 16, 10, 13, 21 and 19, respectively giving a total of 123 genotypes in total (designated collectively as LpFpFam).

### Phenotype evaluations

The list of experimental conditions and traits evaluated are described in Table 1.

**Table 1:** Traits measured across the parental genotypes and LpFpfam.

Environment	Trait
FSC optimum N and P	Tiller number
	Shoot dry weight (dwt)
	Root dwt
FSC low N optimum P	Tiller number
	Shoot dwt
	Root dwt
FSC optimum N low P	Tiller number
	Shoot dwt
	Root dwt
Glasshouse	3 weeks partial flood 10°C–tiller number
	6 weeks partial flood 10°C–tiller number
	3 weeks partial flood 20°C–tiller number
	6 weeks partial flood 20°C–tiller number
	Flood control–tiller number
Glasshouse	Shoot dwt
	Root dwt
	Root length
Drought bins	Recovery after drought (shoot dwt)
	Number reps survived drought period
Outside	Tiller number
	Heading date
	Head number

#### Flowing solution culture

The three trials comparing 1) optimum N and P, 2) optimum N and low P and 3) low N and optimum P were carried out consecutively in the FSC facility at Aberystwyth University. Six tillers of uniform size were taken from established genotypes of LpM, LpL, Fp, S1-S7 and LpFpfam. These were washed free of compost prior to transplantation into FSC (FSC methods were developed and described in [30,35,41–43]. The 6 tillers per genotype were transplanted together in 1litre culture vessels distributed across 8 culture units each containing 200 litres of nutrient solution. Air temperature was controlled at 20/15 ± 1°C day/night, with solution temperatures ambient between 19/20°C throughout the experiment. The plants were grown on under natural light for 20 days after transplant, then supplementary lighting was introduced (10:00–15:00 h), provided by a single 400 W HPI/T lamp (Philips) suspended 1.5 m above the surface of each culture unit (300 ± 25 μmol m^-2^ s^-1^ PAR at canopy height). From day 25 onwards, natural light was excluded and the plants were grown with artificial illumination over a 12 h photoperiod (08:00–18:00 h), provided by paired 400 W HPI/T and SON-T lamps (Philips) above each unit (650 ± 50 μmol m^-2^ s^-1^ PAR at canopy height).

The genotypes were initially given a 28 day ‘nursery’ treatment in order to establish the plants in FSC followed by a further 28 days using 1 of 3 different regimes of N and P: 1) optimum N and P, 2) low N and optimum P or 3) optimum N and low P. For optimum N and P and low N and optimum P, the nursery concentrations of nutrients in the flowing solutions were (μM): NO_3_^-^, 250; K^+^, 250; Ca^2+^, 344; SO_4_^2-^, 424; Mg^2+^, 100; H_2_PO_4_^-^, 50; Fe^2+^, 5.4; with micronutrients as described in [30]. For optimum N and low P, nursery conditions were as described above except that P concentration was reduced to 25 μM. For nutrient treatment conditions, nursery conditions were maintained except for the N and P concentrations which were as follows: optimum N and P, 50 μM N / 50 μM P; low N and optimum P, 2 μM N / 50 μM P; optimum N and low P, 50 μM N / 5 μM P. During treatment periods, nutrients were added daily to each culture unit in a fixed ratio, based on the previously calibrated daily uptake of N by *L*. *perenne* when supplied at 50 μM and 2 μM in this FSC system. A solution pH of 6.0 ± 0.1 was maintained throughout in all units by daily manual additions of 1M H_2_SO_4_.

During the treatment periods, total plant fresh weights per culture vessel (*i*.*e*. bulked for six plants) were recorded non-destructively on days 0, 7, 14, 21 and 28; vessels were randomised weekly across the 8 culture units. All plants were harvested on day 28 and separated into shoot and root fractions. Tiller numbers, shoot and root fresh and dry weights were recorded as bulks of the 6 (or remaining) plants within each vessel. All fractions were oven dried at 80°C for 24 hours prior to measurements of dry weight.

#### Water-logging assays

A pilot experiment was conducted as follows. A tiller of each parental, S1-S7 and LpFpfam genotype was transferred to standard potting compost in a 9cm pot in a glasshouse compartment maintained at 20°C/10°C day/night temperature with supplemental lighting as required to maintain a 10 hour day length. After 4 weeks, the plants were cut back and a small amount of grit overlaid onto the compost to prevent loss of compost during flooding treatments. After a further 4 weeks tiller number was counted (control scores) the plants were cut back to c. 4 cm above the compost surface and the flooding treatment was started. Flooding treatment consisted of partial submergence, in which the water level was maintained at c. 2cm above the top of the compost. Flooding treatments were conducted at two different day temperatures of either 10°C or 20°C for 3 or 6 weeks with a single clone evaluated for each genotype in each treatment. After the end of the treatment period, plants were cut-back to c. 4 cm above the compost surface and tiller number was counted after 2 weeks of regrowth.

Based on the outcomes of this preliminary trial, a second experiment was carried out just using the parental genotypes, S4 and S4fam (omitting a single S4fam genotype which was no longer available). The same general procedure was followed using partial submergence for 3 or 6 weeks, but using 4 replicates of each genotype with plants maintained in a growth room with 20°C/10°C day/night temperature and a 12 hour photoperiod. In addition to tiller number, shoot dry weight was also measured.

#### Drought assays

Drought assays were carried out in drought bins constructed in an unheated glasshouse at ambient temperature. Each drought bin consisted of a 1m x 1.1m x 1.1m (w x l x h) container filled with drainage stone and natural soil to a depth of 40 cm with a top 65 cm layer of potting compost. Five clones of each parental, S1-S7 and LpFpfam genotype were transplanted to randomized positions 7cm apart over 10 drought bins in the autumn and allowed to establish and vernalize over the winter. Edge effects were avoided by using guard rows around the sides of each bin. The bins were irrigated as needed to avoid any water deficit over this period. The following June, after flowering, the soil water content was taken up to field capacity (c. 38%) for one week and the clones were cut-back to c. 4cm above soil level. Water was then withheld for the next 3 months. During this drought period, the night time temperature range varied between 10–20°C and day time temperature range between 15–39°C. After this period, the soil water was taken back up to field capacity and the plants were left to recover for a further 4 weeks. After the end of this period, shoot dry weight and number of surviving clones per genotype were recorded.

#### Shoot and root growth measurements in the glasshouse

Tillers of two replicates of each parental, S1-S7 and LpFpfam genotype were transferred to a mixture of 50:50 vermiculite:grit sand in 15cm square pots with a depth of 20 cm in a glasshouse compartment maintained at 20°C/10°C day/night temperature with supplemental lighting to maintain a 10 hour day length. After 3 months, tiller number, shoot and root dry weight and root length were ascertained.

#### Heading date and head and tiller number measurements outside

Four replicate tillers of each parental, S1-S7 and LpFpfam genotype were established in multipot trays in the autumn and allowed to vernalize in an unheated glasshouse over winter. The following March, each replicate was transplanted to a separate 15 cm pots containing a standard potting compost and randomised on an external, hard-standing area. Heading date (emergence of 3 heads), tiller number on the 1st May and number of heads on the 16th June were recorded for each genotype.

### Genotyping

LpM, LpL, Fp, S1-S7, S1fam1-S7fam (n = 8, 13, 23, 16, 10, 13 and 21, respectively) and LpCon family (n = 19) were genotyped using the Illumina Infinium SNP genotyping microarray [44]. DNA samples used for genotyping templates were extracted from leaf tissue using the DNeasy Plant Mini Kit (Qiagen Ltd, Manchester, UK).

### Marker/trait associations

Marker/trait associations were established using one-way analysis of variance (ANOVA) or a Kruskal-Wallis test (KW) on a marker-by-marker basis across all of the genotypes in LpFpfam, with Bonferroni correction for family-wise error rate, and implemented using Genstat for Windows 18th Edition. Associations were only considered to be significant if the p-value for the marker-trait association was less than this threshold. The chromosomal positions of markers were inferred from genetic maps previously constructed using the Lolium Illumina Infinium SNP genotyping array using a *L*. *perenne* pseudotestcross mapping population (AberMagic x Aurora) and a *L*. *perenne* F_2_ mapping population [45].

### *F*. *pratensis* introgression

#### Trait associations with Fp-derived chromosome segments

Trait data were also analysed in terms of the mean scores for all of the genotypes containing a defined segment of *F*. *pratensis* introgression, using the recombination bins previously described to define common *F*. *pratensis* introgressed segments shared between genotypes [22].

#### Generic consequence of *F*. *pratensis* introgression

The relative sizes of the individual *F*. *pratensis* introgressions in this family based on G*IS*H discrimination of metaphase chromosomes has been previously reported [22]. Based on this report, the relative degree to which the *L*. *perenne* genome had been substituted by the *F*. *pratensis* genome (as illustrated in S1B Fig) in each individual was evaluated against trait performance across S1fam1-S7fam using Spearman’s rank correlation.

## Results

### SNP genotyping

From the total number of 3541 potential marker assays on the SNP array, 976 markers were identified that were clearly segregating across the 123 genotypes within LpFpFam and had been assigned map positions in either of the AberMagic x Aurora or F_2_ mapping populations. The minimum number of genotype scores for any marker was 58, and the majority of markers (68%) were scored across all 123 genotypes within LpFpFam (mean = 121.8). Across all 976 markers and 123 genotypes, of the 120048 possible genotype calls, c. 45.5% of the genotypes were scored as heterozygous, c. 53.5% as homozygous and c. 1% were null.

### Marker trait associations

Significant marker trait associations identified using ANOVA or KW on a marker-by-marker basis with Bonferroni correction are summarized in Table 2. Probabilities and genetic map positions of significant markers are provided in S1 Table, and probabilities for all marker trait associations and genetic map positions are provided in S2 Table.

**Table 2:** Derived chromosomal regions containing significant markers for indicated traits.

LG2^a^			LG3			LG5		
Trait^b^	F_2_ (cM)	AxA (cM)	Trait^b^	F_2_ (cM)	AxA (cM)	Trait^b^	F_2_ (cM)	AxA (cM)
Ro_Np	60–64	51–66	Ro_NP	-	13	Ro_NP	-	53
Hd	22–63	20–51	Ro_Np	-	13	-	-	-
-	-	-	Ro_nP	46–47	22–24	-	-	-

^a^LG2/3/5 = linkage groups 2, 3 or 5 in the *L*. *perenne* F_2_ or AberMagic x Aurora (AxA) genetic maps. Numbers in F_2_ and AxA columns indicate the ranges of the genetic map positions in centiMorgans (cM) of markers significantly associated with the indicated traits on that linkage group.

^b^Trait key: **NP** = optimum N and P; **nP** = low N, optimum P; **Np** = optimum N, low P in flowing solution culture. **Ro** = root dwt; **Hd** = heading date.

Significant marker trait associations were identified for root biomass and heading date. None were identified for tiller number, shoot biomass, plant height and root length or in response to any of the waterlogging or drought evaluations. Of particular note were: 1) root biomass was associated with regions on chromosome 3 (C3) in all FSC nutrient conditions and, 2) a region on chromosome 2 (C2) was also significantly associated with root biomass, but specifically in optimum N and low P FSC conditions. For this latter case, the alleles on C2 associated with enhanced performance in optimum N and low P were derived from the LpM parent with LpM-derived allelic variation for marker Contig38631_269 (the most significant marker/trait association; S1 Table) resulting in c. 78% increase in root dry weight relative to genotypes under the same conditions without this LpM-derived allele. LpM itself did not survive under low N and optimum P conditions and grew poorly under optimum N and P relative to Fp and LpL. However, while LpM still produced 25–50% less biomass than Fp and LpL under optimum N and low P conditions, its growth was enhanced 2.5–3 times in terms of shoot and root biomass, relative to its growth under optimum N and P. It is likely that this relative enhancement of growth of LpM under optimum N and low P conditions has the same genetic basis as the significant region of marker/trait association on C2 with root biomass which is associated with segregation of LpM-derived alleles in LpFpFam.

A further significant marker-trait association for root biomass was identified on chromosome 5 (C5). While only this trait was significant after Bonferroni correction, this region (specifically marker Contig50116_879; 33cM, Abermagic x Aurora genetic map) was associated significantly with a number of independently evaluated traits before multiple testing correction (*i*.*e*., different FSC conditions, glasshouse, flooding and drought trials; S3 Table). So this region of C5 may have more general effects in enhancing plant performance.

### Correlations between trait scores

To ascertain whether the general response of genotypes were comparable between FSC and the container-based measurements, trait scores across LpFpfam were compared on a pairwise basis for correlation. Multiple significant positive correlations were identified both within and between independent trials, indicating consistent trends in relative growth performances across FSC and in the container-based measurements (see S4 Table). In addition, a number of negative correlations were associated with heading date, indicating that there was trend for genotypes with later flowering to have fewer tillers and flowering heads.

### *F*. *pratensis* genome introgressions and trait variation

#### Introgressed chromosomes and segments

**Fig 1:**
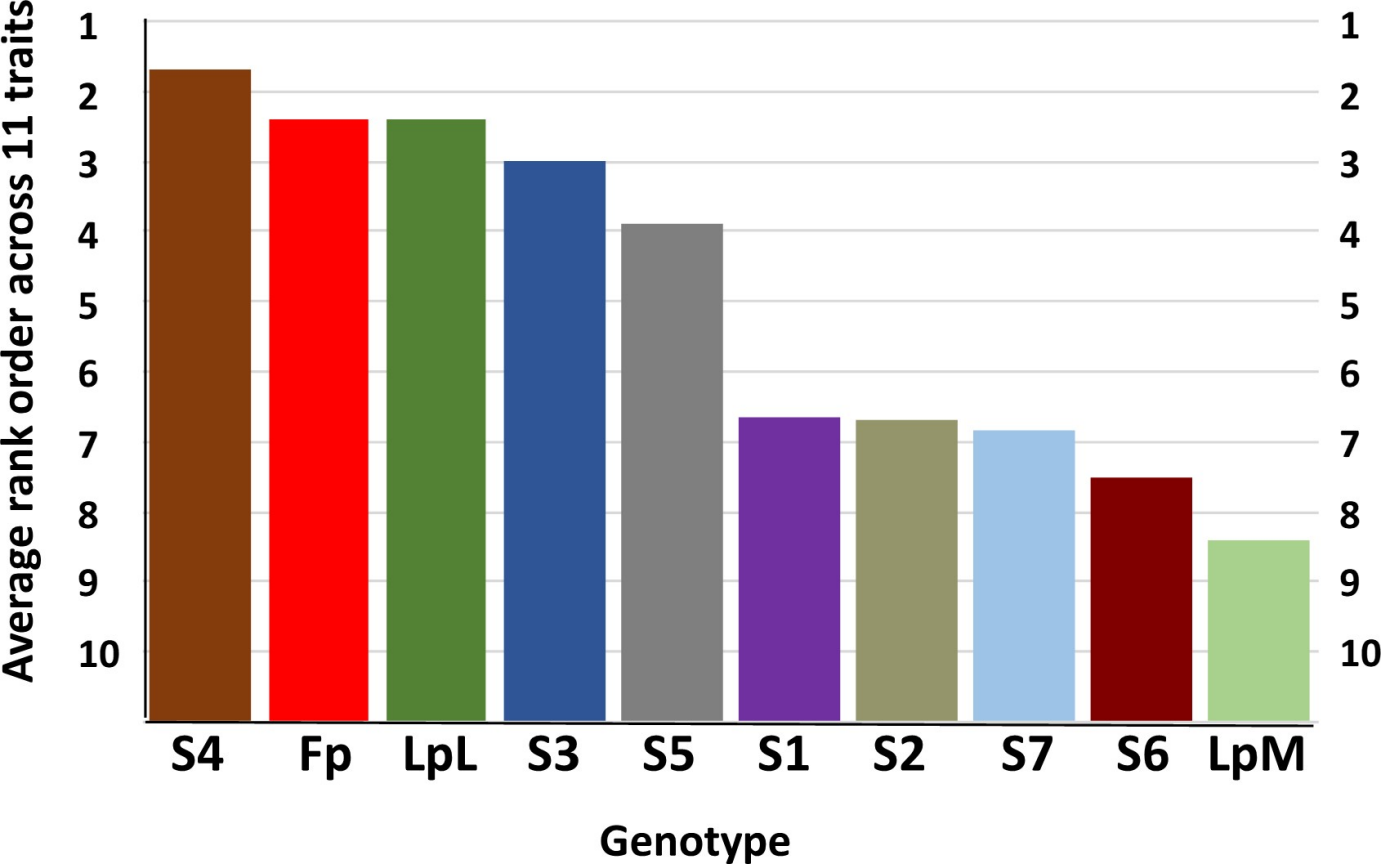
Average rank orders across 11 independently measured traits for Fp, LpM, LpL, and S1-S7. Traits measured were shoot dry weight in FSC optimum N and P, optimum N and low P, low N and optimum P (Table 1, Environments 1, 2 and 3); tiller number in partial flooding at 10°C and 20°C after 3 and 6 weeks in glasshouse conditions, and 20°C with no flooding Table 1, Environment 4); shoot dry weight in drought bins (Table 1, environment 6); shoot dry weight in glasshouse (Table 1, Environment 5); tiller number outside (Table 1, Environment 7). For each of the 11 trait measurements, genotypes were ranked from 1–10 in terms of shoot dry weight or numbers of tillers, with the genotype with the largest shoot dry weight or greatest number of tillers scored as ‘1’, and the average rank order derived.

**Fig 2:**
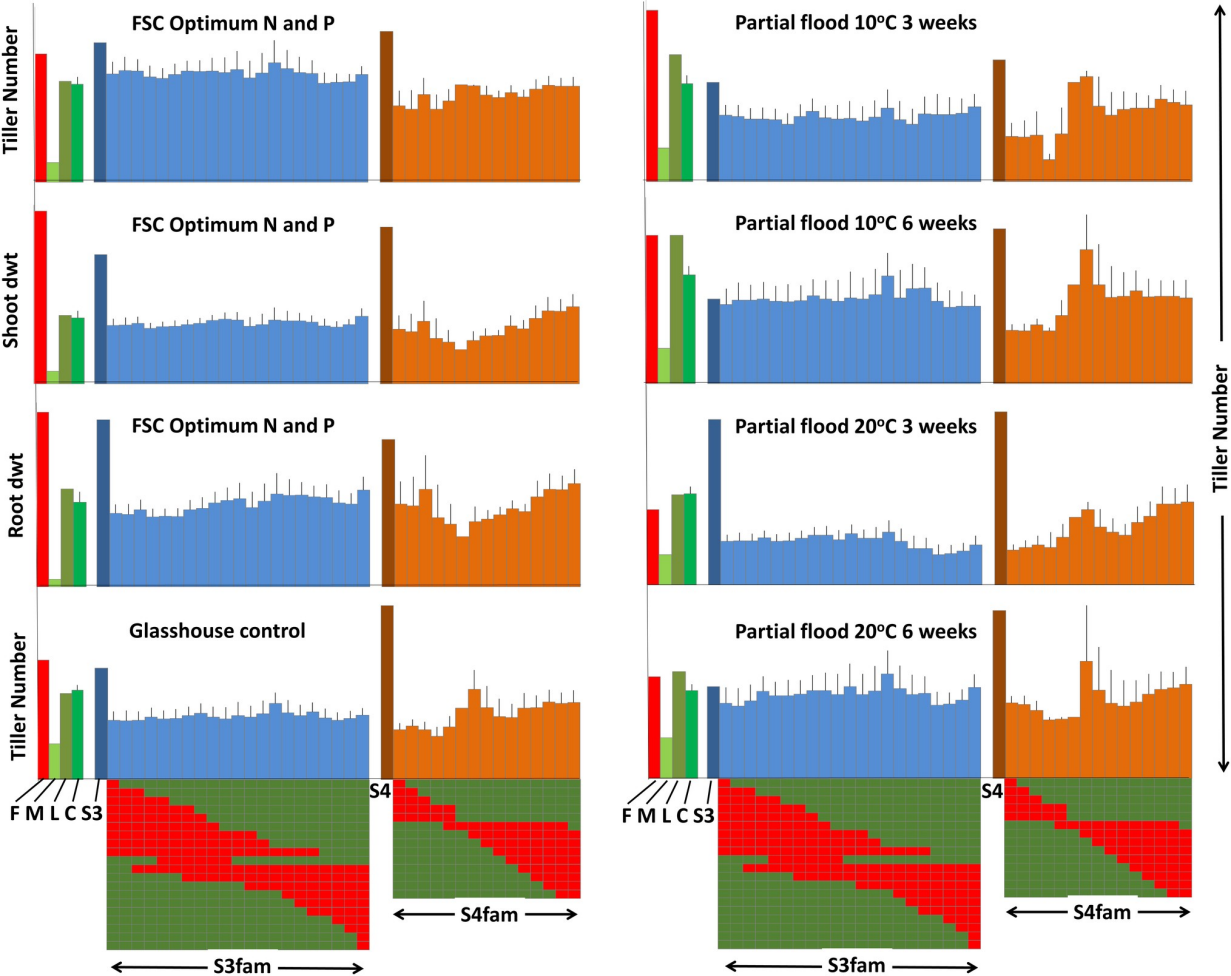
Relative trait variation across Fp, LpM, LpL, LpCon, S3, S4, S3fam and S4fam for selected trials. For Fp (F), LpM (M), LpL (L) S3 and S4 columns represent the score for the individual genotype; for LpCon (C), the column represents the mean of the LpCon population; for S3fam and S4fam, each column in each graph is the mean of the trait score for the genotypes within S4fam which contain a *F*. *pratensis* introgression at that particular position (illustrated below the *x* axis). For each illustration of the pattern of *F*. *pratensis* introgressions, each row represents a single genotype and each column a defined and sequential region of the chromosome from end to end. A red square indicates a *F*. *pratensis* introgression at that relative position in that particular genotype; a green square indicates the *L*. *perenne* background. Error bars are standard errors (n<2) or the range (n = 2).

**Fig 3:**
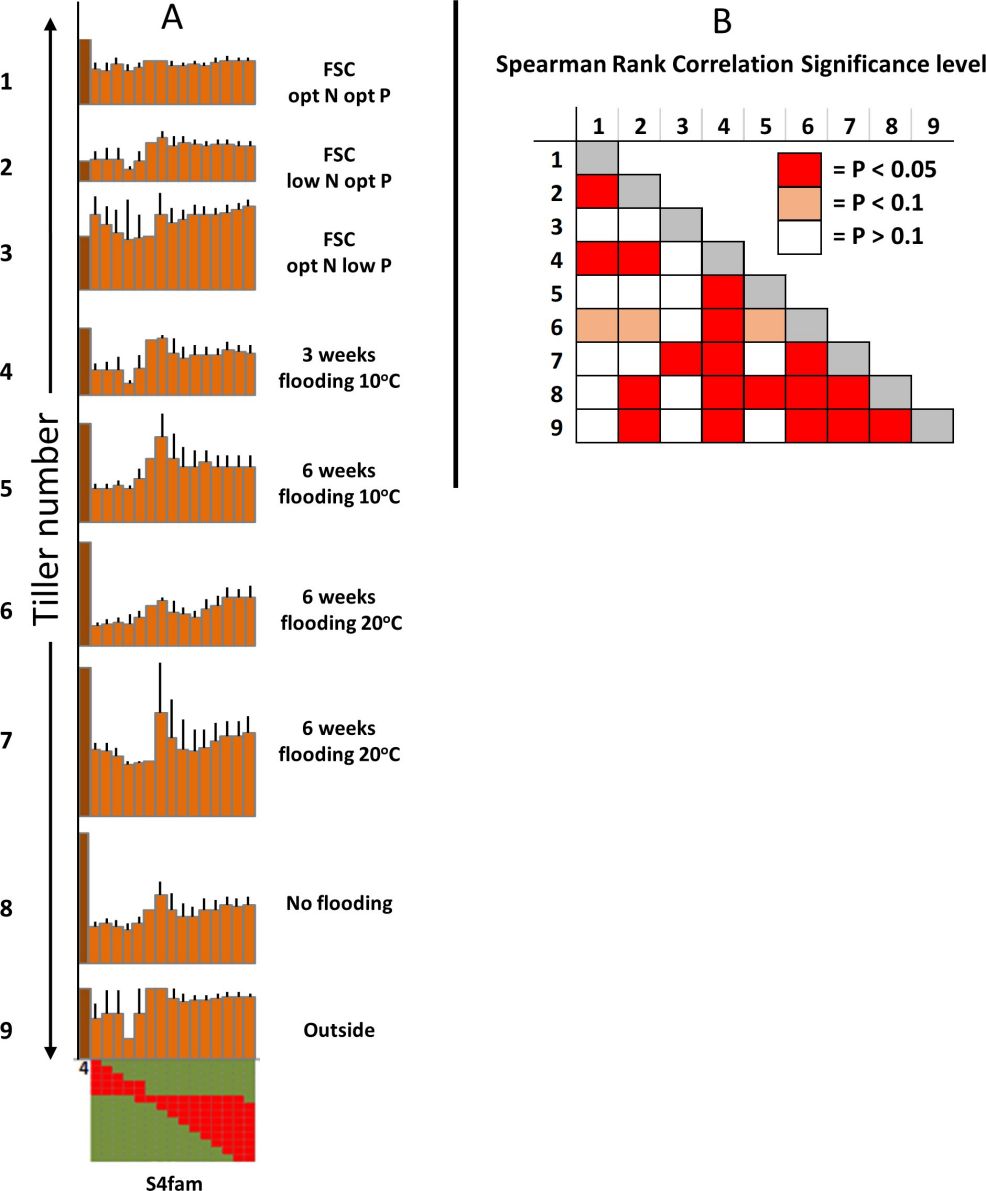
**A.** Relative variation in tiller number within S4fam represented according to the position of the introgressed *F*. *pratensis* chromosome segment. Each column in each graph is the mean of the trait score for the genotypes within S4fam which contain a *F*. *pratensis* introgression at that particular position (illustrated below the *x* axis). For each illustration of the pattern of *F*. *pratensis* introgressions, each row represents a single genotype and each column a defined and sequential region of the chromosome from end to end. A red square indicates a *F*. *pratensis* introgression at that relative position in that particular genotype; a green square indicates the *L*. *perenne* background. Error bars are standard errors (n<2) or the range (n = 2). **B.** Spearman rank correlation significance levels for individual genotype tillering scores in S4fam from the 9 trials described in Fig 3A. Numbers refer to the numbering in Fig 3A.

Table 1 details the list of measurement that were recorded across LpM, Fp, LpL, S1-S7 and LpFpfam to examine the relationship of specific *F*. *pratensis*-derived introgressions to trait performance in this *L*. *perenne* background. When genotypes LpM, Fp, LpL and S1-S7 were ranked for 11 independent measures of shoot dry weight or tiller number (*i*.*e*., different trials, different clones, using either shoot dry weight or tiller number and not root dry weight or length), S4 was the strongest genotype and LpM was the weakest (Fig 1). S4 and S3 were similar to Fp and LpL, S5 was more intermediate and S1, S2, S6 and S7 were more similar to LpM. The one exceptional trait was regrowth after water stress in the drought bins, in which the LpL parent far outperformed all of the other genotypes (see S2C Fig). To investigate the degree to which we could detect whether positive trait performance differences might be attributable to particular genomic regions inherited from the Fp parent, trait performance was evaluated from the perspective of the inheritance of particular sub-chromosomal introgressed segments generated from the further crosses to LpL (S1A Fig). Focusing on S3, S4 and their derived progenies, Fig 2 illustrates S3fam and S4fam scores, described according to sub-chromosomal introgression, for trials for which S3 and S4 had strong trait scores. While no particular trends were apparent across the S3fam *F*. *pratensis* introgression series, a consistent trend was observed across the S4fam introgression series, dependent on which end of the *F*. *pratensis* chromosome 4 (C4) was present. Fig 3 illustrates this association between particular Fp-derived introgressions on C4 and all scores of tiller number—each of the traits scores illustrated were taken from independent evaluations. It can be seen that there was a trend for genotypes with introgressions at one end of C4 to produce fewer tillers than genotypes with introgressions at the other end of C4. Of the 36 rank order pairwise correlations between genotype scores for the independent assessments of tillering illustrated in Fig 3, 18 were positively correlated with p<0.05 and a further 3 with p<0.1 –indicating that genotype performance was relatively consistent across the independent trials.

**Fig 4:**
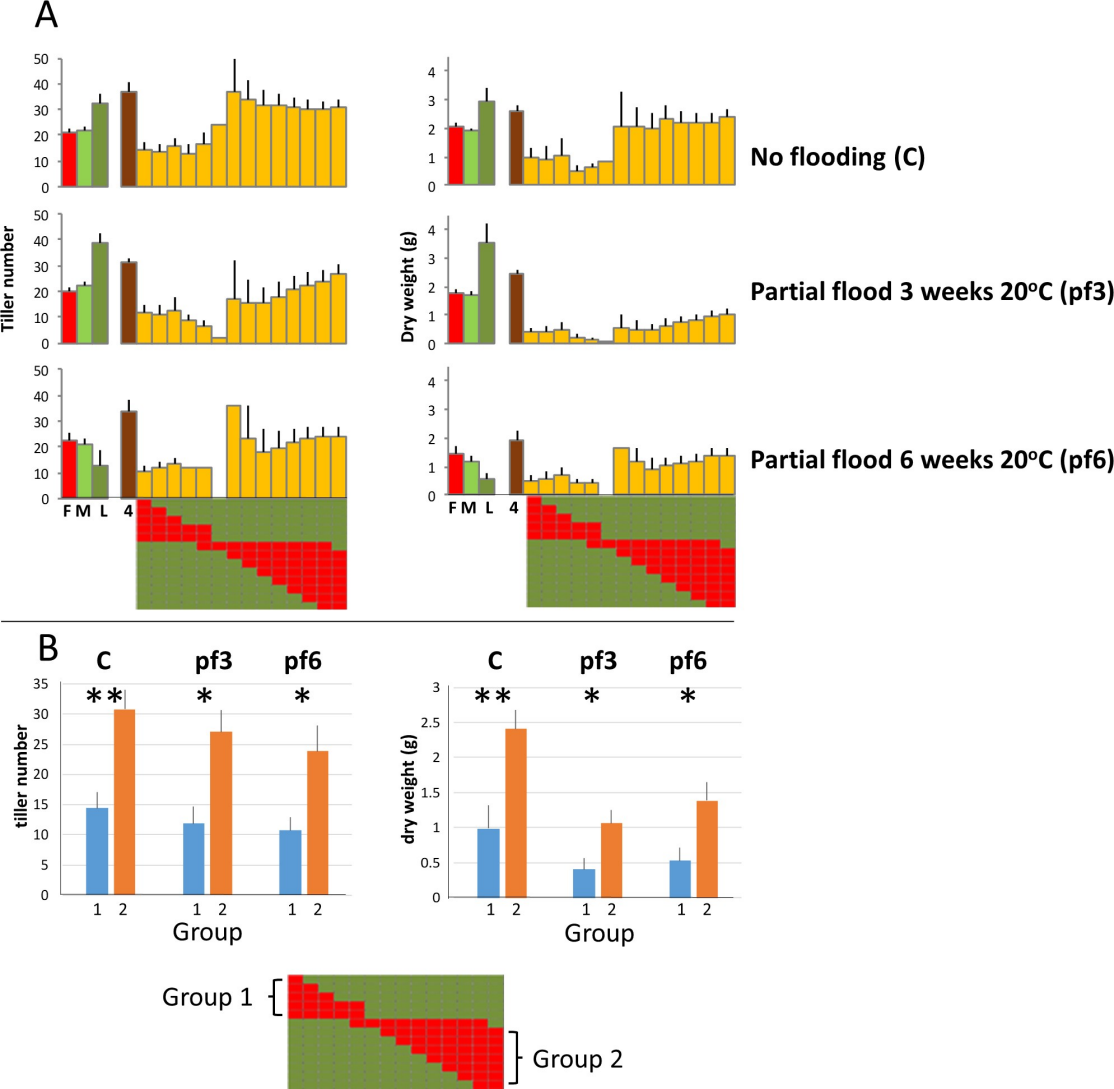
**A**. Relative variation in tiller number within S4fam measured in quadruplicate in partial flooding and control conditions, represented according to the position of the introgressed *F*. *pratensis* chromosome segment. Each column in each graph is the mean of the trait score for the genotypes within S4fam which contain a *F*. *pratensis* introgression at that particular position (illustrated below the *x* axis). For each illustration of the pattern of *F*. *pratensis* introgressions, each row represents a single genotype and each column a defined and sequential region of the chromosome from end to end. A red square indicates a *F*. *pratensis* introgression at that relative position in that particular genotype; a green square indicates the *L*. *perenne* background. Error bars are standard errors (n<2) or the range (n = 2). **B.** Variation in tiller number in flooding trials for genotypes within S4fam with *F*. *pratensis* introgressions at different ends of C4 (Groups 1 and 2). * and ** = significant at p = 0.05 and 0.01, respectively.

In order to confirm this trend concerning *F*. *pratensis* introgressions on C4, a further trial was carried out measuring tiller number and shoot dry weight under control and partial flooding conditions for parental, S4 and S4fam genotypes, measured in quadruplicate in controlled environment growth rooms. The results, illustrated in Fig 4, show this same pattern with significant differences for both tiller number and shoot dry weight for groups of plants possessing opposite terminal Fp introgression segments.

#### Generic effects of introgression

Considering genotypes within S1fam-S6fam (*i*.*e*., those genotypes for which the crossing scheme involved a cross followed by a single backcross to LpL–see S1A Fig) the correlation between the estimated percentage of the genome represented by *F*. *pratensis* introgression and all 21 of the measured vegetative growth-related phenotypes was negative, with p < 0.05 for 11 of these negative correlations (S5 Table), including all 5 of the independent measurements of root growth. If all genotypes in S1fam-S7fam were included (production of S7fam involved a second backcross to LpL; S1A Fig), the direction of correlation was also negative for all of the 21 vegetative growth-related phenotypes, with 7 being significant (p < 0.05) four of which were root traits (S5 Table). The only positive correlations were with heading date (*i*.*e*., later flowering times) and both were non-significant (p = 0.08 and 0.12).

## Discussion

This *L*. *perenne* x *F*. *pratensis* festulolium introgression population was originally developed as a method for comparative genomic mapping and exploited the fact that, while these species could be distinguished readily in terms of genetic polymorphism, G*IS*H, morphological features and physiological responses, at the same time they shared similar karyotypes and showed frequent interspecific chromosome recombination at meiosis. Thus, genetic recombinants could both be generated and distinguished relatively easily and major effects of interspecies chromosome recombination on phenotype might become apparent. King et al. [22] reported the results of the comparative genomic mapping in this festulolium family and, in this study, we have followed this up with a characterization of some aspects of the trait biology of this population in relation to the underlying genetics/introgression genomics. In this latter context, while being ideal for comparative genetic and genomic mapping, this population has presented some challenges in terms of the genetic analysis. For instance, while the gene pool for all 130 of the genotypes which comprised S1-S7, LpFpfam and LpCon was derived from the same 3 genotypes (Fp, LpM and LpL), the fact that S1-S7 (the genotypes containing the 7 different monosomic introgressions) all had to be back-crossed separately with LpL meant that the segregation patterns for individual markers varied across the 7 families of which LpFpfam was constituted. While this did not affect the development of individual marker-trait associations, it did mean that assigning markers to relative regions within the genome was accomplished using orders and positions for these specific markers derived from previous *L*. *perenne* genetic mapping studies, rather than directly through genetic mapping within the population.

In terms of the specific choice of the LpM and LpL genotypes from the varieties Meltra and Liprior, this was partly due to the experimental tractability of these genotypes for developing systematic introgression populations–*i*.*e*., the ability to isolate the fertile triploid and then derive the initial 7 monosomic substitution lines relatively easily from crosses of these genotypes. In fact, other combinations of tetraploid and diploid *L*. *perenne* (and *F*. *pratensis)* genotypes were attempted, but low or absent fertility was a common feature. However, we were also aware that the cross represented a) an early x late heading date combination and b) a genetic background that had been selected in the development of a tetraploid variety with a genetic background that had been selected in the development of a diploid variety. So, this might be an interesting cross *per se* in terms of trait expression.

### Marker-trait associations

Meltra is a late flowering variety and Liprior is an early flowering variety, therefore it is not surprising that significant marker-trait associations with heading date were identified, all located on C2. C2 has been reported as containing heading-date QTL in a number of previous bi-parental and GWAS studies [46–49] and while the lack of common markers across studies makes aligning QTL problematic, the position of the significant marker-trait associations in the Meltra x Liprior cross reported here is not incompatible with the position of the heading date QTL detected in the *L*. *perenne* F_2_ family, also an early x late heading date cross; [46] (one of the families used for allocating genetic map positions in the present study). The remainder of the chromosomal regions with significant marker-trait associations were all for root dry weight in FSC. These were present across all of the FSC conditions on C3, on C2 in optimum N and low P, and on C5 in optimum N and P. One reason that these findings are of generic interest is that reports which provide resolution at the levels of specific markers or QTL in perennial ryegrass relating to root growth are rare due to the obvious experimental challenges in obtaining the necessary measurements. While there are a number of reports of QTL identification for different aspects of root growth from the cereals which, across different studies, have assigned QTL to all 7 of the Triticeae homoeologous chromosome groups [50–54], with similar findings in rice [52,55–62], most of the studies that have looked at root growth in perennial ryegrass have been population-based studies of root biomass [63–67]. Importantly, our findings establish that, as in cereals and rice, there is potential for selecting for root growth traits in perennial ryegrass if the right assays and populations are in place.

Comparing the parental *L*. *perenne* genotypes LpM (Meltra) and LpL (Liprior), LpM produced markedly less root growth in FSC under all nutrient conditions than LpL, though performed relatively better under optimum N and low P than under optimum N and P or low N and optimum P. Comparable differences were apparent in the measurements of root growth made under glasshouse conditions, where LpM produced approximately a third of the total root biomass of LpL and approximately one half of the total root length (the same trend, though less marked, was also apparent for shoot dry weight–though marker-trait associations were above the level of significance for this trait). It is possible to distinguish the genetic effects influencing root dry weight identified on C2 and C3. The C2 region (most significant marker Contig38631_269) was only detected in FSC under optimum N and low P nutrient conditions and increased root dry weight was associated with the presence of an allele derived from LpM. For the C3 region (most significant markers: Contig8711_2766, optimum N and P, optimum N and low P; Contig31158_1560, low N and optimum P) increased root dry weight was associated with the presence of alleles derived from the LpL parent. The fact that the root growth region on C2 was specific to the P-limited conditions is of particular interest. It is well documented that future sources of P for plant fertilizer are potentially limited [68] and it is a key aim of grass breeders to be able to reduce P and other inputs while still retaining acceptable yields and delivering environmental services. Enhanced root growth under lower P and N regimes would clearly be an important component of this. Numerous studies in cereals have indicated that P-use-efficiency is a complex trait with multiple QTL contributing to the overall response, and root growth will only be one component of this response [69–79]. Thus, the transfer of this region of C2 into different ryegrass and festulolium backgrounds will allow us to further assess its potential. Additionally, future fine-mapping of this region, combined with transcriptomic studies to identify candidate genes (*e*.*g*., high affinity phosphate transporters are known to be upregulated in *L*. *perenne* roots in response to P-limitation [80] and important in P-uptake in other grass species [81–84]) should be revealing. A further region on C5 was significantly associated with root growth in optimum N and P. Within this region, marker Contig50116_879 was also significantly associated with a wider range of independently measured traits across a number of the conditions on the basis of the uncorrected probability value (p < 0.05). For root dry weight in FSC in low N and optimum P conditions and for glasshouse grown plants, these probabilities were significant within a 5% Benjamini-Hochberg false discovery rate ([85]; S3 Table). It would be interesting to know whether the enhancement of trait performance across a number of nutrient and abiotic stress conditions associated with this region on C5 is a direct consequence of differential root development or whether this region has more generic effects on whole plant growth responses. Certainly, C5 has been identified as containing QTL for responses to a number of abiotic stresses (winter survival, freezing tolerance, herbage survival and regrowth after water stress [86,87] but lack of common markers means that more than very general alignments between studies are not possible.

### Evaluating the effects of *F*. *pratensis* introgressions

The *F*. *pratensis* genotype chosen for the present study was derived from a seed accession of a Russian landrace. At the time when the initial crosses were made to derive S1-S7, a combination of the experimental usefulness of this genotype, in terms of its utility within the crossing scheme, along with glasshouse and field-based observations that it was a robust genotype in the circumstances in which it was to be grown, suggested that specific *F*. *pratensis* introgressions from Fp might have significant influences on phenotype within the Meltra/Liprior *L*. *perenne* background. This can be placed in the context of a number of studies where it has been possible to link *Festuca* spp. introgressions with defined traits [3–5,17,18,21,88–90]. Following on from this, after isolation of all 7 of the *F*. *pratensis* monosomic introgression lines, S1-S7 [25] used in the present set of experiments, it was apparent that S3 and S4 were vigorous genotypes in comparison to the remainder of the monosomic introgressions (Mike Humphreys, personal communication). In the present study we sought to re-examine S1-S7 phenotypes to see if the differences in growth responses between S3 and S4 and the remainder of the monosomic introgressions were stable across different experimental conditions and if any observed effects might be directly attributable to the introgression of a gene(s) present on a particular alien chromosome segment–a finding which would have potential application in festulolium breeding.

In the studies presented here, S3 and S4 had high trait scores under many of the conditions tested (Fig 2). In fact, when trait performance of S3 and S4 is illustrated according to rank order across the 11 independent evaluations described in Fig 1, but in comparison to all of the genotypes used in these experiments (*i*.*e*., Fp, LpM, LpL, S1-7 and LpFpfam) S4 was ranked 4^th^ and S3 21^st^ out of 138 (S3A Fig). What is surprising about this is that S1-S7 contain a higher proportion of LpM derived alleles (c. 50%) compared to LpFpfam genotypes which contain c. 25% LpM alleles (S1A Fig). As LpM was ranked 135^th^ out of 138 one might expect S3 and S4 to have mid-range to lower rank scores–as do S1, S2, S5, S6 and S7 (S3A Fig); in fact, S4 was ranked above both LpL and Fp. It would be interesting to take this further and test these observations for consistency by generating a number of examples of each of the monosomic introgressions in different combinations of the LpM/LpL backgrounds. Unfortunately, the resources required to develop such populations militate against this being a practical option.

However, when looking at trait performance of S4fam genotypes, there was evidence of variation in trait scores across genotypes dependent on which end of Fp-derived C4 had been transferred to the progeny (Figs 3 and 4) with the average ranks of S4fam Group1 and Group 2 genotypes (see Figs 3 and 4) being 104 and 44, respectively (S3B Fig); S4 and S4fam Group 2 genotypes contributed 5 out of the top 14 genotypes (top c. 10%) in terms of their rank orders across the 11 independently measured traits. Interestingly, this effect of trait enhancement associated with one end of Fp C4 could be seen irrespective of the magnitude and relative trait scores of LpM, LpL, Fp or S4 –so it is a more general effect on plant vigour rather than, obviously, a direct transmission of a particular phenotype from the Fp parent. In contrast to S4fam, there was no evidence that any enhanced trait performance seen in S3 (see Fig 2) had been transmitted to S3fam *via* a specific Fp-derived chromosomal region. Following on from this in the context of festulolium breeding, using parental introgression genotypes/phenotypes, such as seen in S3 and S4, to predict progeny performances will not necessarily be straightforward. A challenge of the outcomes of the present study will be to test the effects of introgression of the Fp C4 chromosomal region which enhanced trait performance in this *L*. *perenne* background, in current pre-breeding germplasm selections.

It is, perhaps, surprising that amongst the genome-wide set of sub-chromosomal introgressions that have been analysed within LpFpfam, there is not more evidence of either positive, negative or even disruptive effects on phenotype. This may just reflect the close evolutionary distance between *L*. *perenne* and *F*. *pratensis* (their most recent common ancestor is estimated variously at between 1 and 7 million years ago [91–94]) and the ability to discriminate these species’ genomes using G*IS*H is not a direct measure of similarity of the genic content and may give an artificially high impression of their diversity. In fact, natural hybrids are known to occur and the ryegrass and fescue genomes have combined over evolutionary time to form a widely distributed polyploid series–indicating a broad intergenomic compatibility. It is, however, worth noting that one region of the *F*. *pratensis* genome on C6 could not be recovered as a recombined segment (S1B Fig) and while the whole chromosome substitution within S6 was identified, this was only after prolonged screening (unlike S1-5 and S7 which were identified relatively easily; John Harper, unpublished). So, it is likely that there is some selection against generation, transmission or viability of this *F*. *pratensis* genomic region in this *L*. *perenne* background (discussed in general in [95,96]).

A secondary observation can be made relating to just the relative percentage of the whole genome represented by *F*. *pratensis* introgression. The relative sizes of the individual *F*. *pratensis* introgressions in this family based on G*IS*H discrimination of metaphase chromosomes has been previously reported [22]. Extrapolating from this report, we can estimate the proportions of the whole genomes within each of the S1fam-S7fam genotypes in which the *L*. *perenne* background has been substituted by *F*. *pratensis*. Considering just genotypes within S1fam-S6fam (*i*.*e*., those genotypes for which the crossing scheme involved a cross followed by a single backcross to LpL–see S1A Fig) with the exception of heading date, the correlations between the estimated percentages of the genome represented by *F*. *pratensis* introgressions and the measured phenotype were negative, with p < 0.05 for 11 of these negative correlations—including all 5 measures of root growth (S5 Table). If all genotypes in S1fam-S7fam are included, the direction of correlation is the same for all traits–though only 7 are significant (the production of the S7fam genotypes involved a second backcross to LpL and this will have affected the relative proportions of the genome contributions of LpM and LpL in the *L*. *perenne* background for S7 and derived progeny; S1A Fig). What the biological basis for this preponderance of negative correlations associated with the percentage of the genome composed of introgressed *F*. *pratensis* segments is unclear, it is the opposite of what one might expect if heterosis was a significant factor. Possibly, *F*. *pratensis* introgression into *L*. *perenne* also involves the introduction of weakly deleterious (for *L*. *perenne*) *F*. *pratensis*-derived alleles, the number proportional to the relative size of the introgressed *F*. *pratensis* segment. Following from this, in the absence of any major and counteracting beneficial effects of introgression, this might manifest in marginally reduced vigour. It would be very interesting to know if this low-level ‘debilitating’ effect is generic in terms of *L*. *perenne*/*F*. *pratensis* introgression or family specific, as this may have implications for the selection of parental genotypes and the design of crossing schemes in festulolium plant breeding programmes. As in exploiting heterosis in inbreeding species, evaluations of combining ability between prospective *L*. *perenne* and *F*. *pratensis* parents may come to play an important role in festulolium improvement.

## Conclusion

We have identified genetic effects on root growth segregating in the Meltra x Liprior *L*. *perenne* background on C2, C3 and C5. The effect on C2, inherited from the LpM parent, was specific to P-limited conditions. These results suggest avenues for developing experimental populations and germplasm resources for understanding and improving root growth and P-use-efficiency in forage grasses.

Two major effects were associated with the genome-spanning *F*. *pratensis*-derived introgressions evaluated across this family: 1) the monosomic introgression for *F*. *pratensis* C4 and a derived sub-chromosomal region at one end of the chromosome were associated with enhanced plant growth across a number of the test conditions; and 2) there was a negative correlation between the general extent of *F*. *pratensis* genome introgression and trait expression–particularly for root growth. These results, indicate that *F*. *pratensis* introgression into the *L*. *perenne* background can have positive effects on quantitative traits but there may be co-incident challenges associated with predicting progeny performances from parental introgression genotypes and, more generally, low-level negative consequences associated with the extent of alien chromosome introgression.

## Supporting information

S1 Fig **A. Crossing scheme for pairs of genotypes used to generate LpFpfam. B. Diagrammatic representation of the single chromosomes carrying the F. pratensis introgressed segments in the L. perenne background**.
(PPTX)

S2 Fig Trait variations across the parental genotypes and S1fam-S7fam. (PPTX)

S3 Fig Average rank order for the parental genotypes and LpFpfam across 11 independently measured traits. (PPTX)

S1 Table Probability values for marker trait associations significant after Bonferroni correction. (XLSX)

S2 Table Probability values for all marker traits associations across LpFpfam. (XLSX)

S3 Table Significant associations between C5 marker Contig50116_879 and indicated traits. (DOCX)

S4 Table Probability scores for Spearman's rank correlation tests between all pairs of phenotype assays conducted on LpFpfam. (XLSX)

S5 Table Correlations between trait scores and the estimated percentage of the *F*. *pratensis* genome introgressed into each genotype. (XLSX)
