# Supplementary material for: An investigation of genotype-phenotype association in a festulolium forage grass population containing genome-spanning Festuca pratensis chromosome segments in a Lolium perenne background
Source: PLoS One. 2018 Nov 14;13(11):e0207412. doi: 10.1371/journal.pone.0207412 (PMC6235365; doi:10.1371/journal.pone.0207412)
Supplement: S1 Fig — A. Crossing scheme for pairs of genotypes used to generate LpFpfam. B. Diagrammatic representation of the single chromosomes carrying the F. pratensis introgressed segments in the L. perenne background. (PPTX) [file pone.0207412.s001.pptx]

## Slide 1
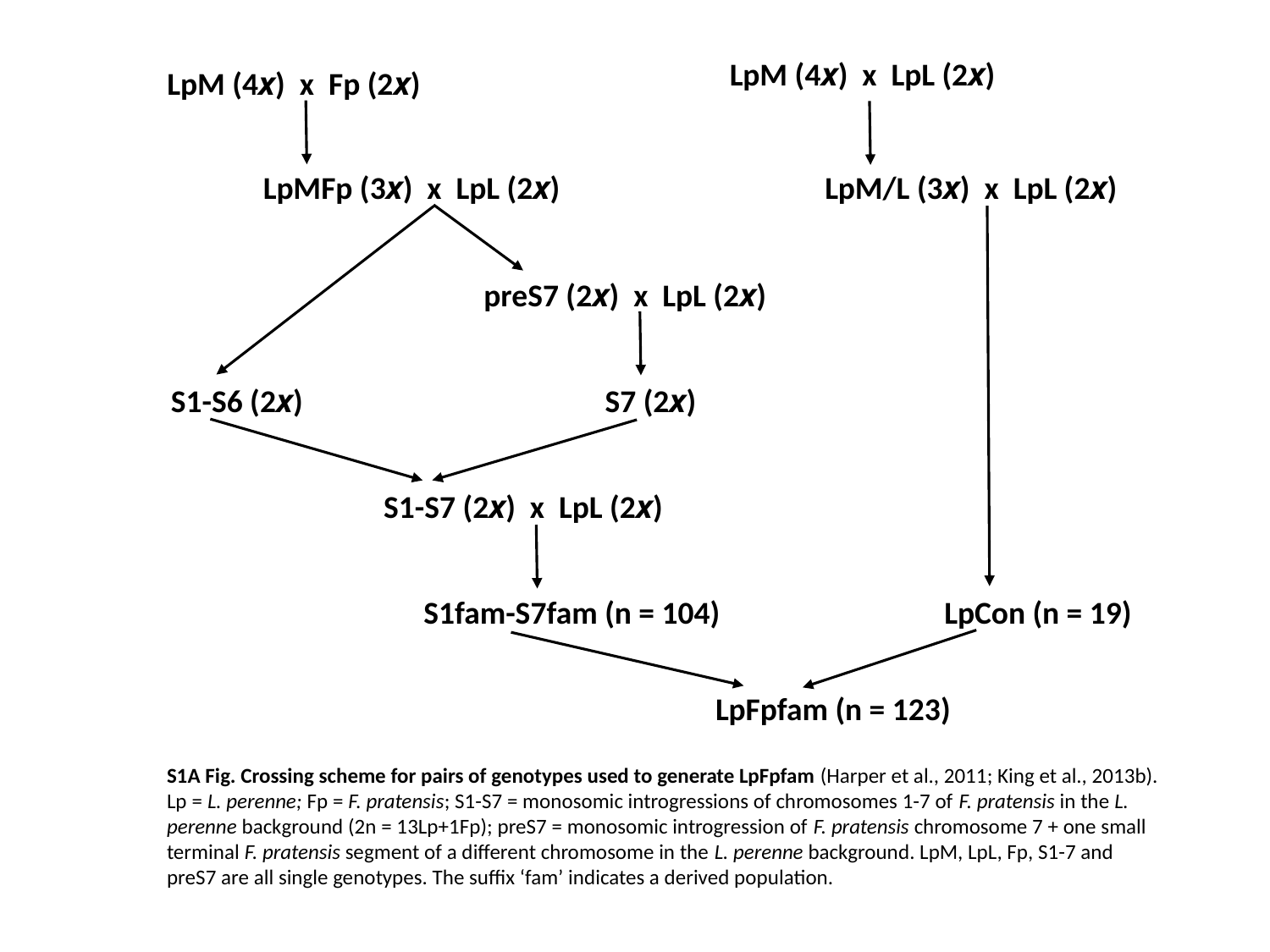

LpM (4x) x LpL (2x)
LpM (4x) x Fp (2x)
LpMFp (3x) x LpL (2x)
LpM/L (3x) x LpL (2x)
preS7 (2x) x LpL (2x)
S1-S6 (2x)
S7 (2x)
S1-S7 (2x) x LpL (2x)
S1fam-S7fam (n = 104)
LpCon (n = 19)
LpFpfam (n = 123)
S1A Fig. Crossing scheme for pairs of genotypes used to generate LpFpfam (Harper et al., 2011; King et al., 2013b). Lp = L. perenne; Fp = F. pratensis; S1-S7 = monosomic introgressions of chromosomes 1-7 of F. pratensis in the L. perenne background (2n = 13Lp+1Fp); preS7 = monosomic introgression of F. pratensis chromosome 7 + one small terminal F. pratensis segment of a different chromosome in the L. perenne background. LpM, LpL, Fp, S1-7 and preS7 are all single genotypes. The suffix ‘fam’ indicates a derived population.

## Slide 2
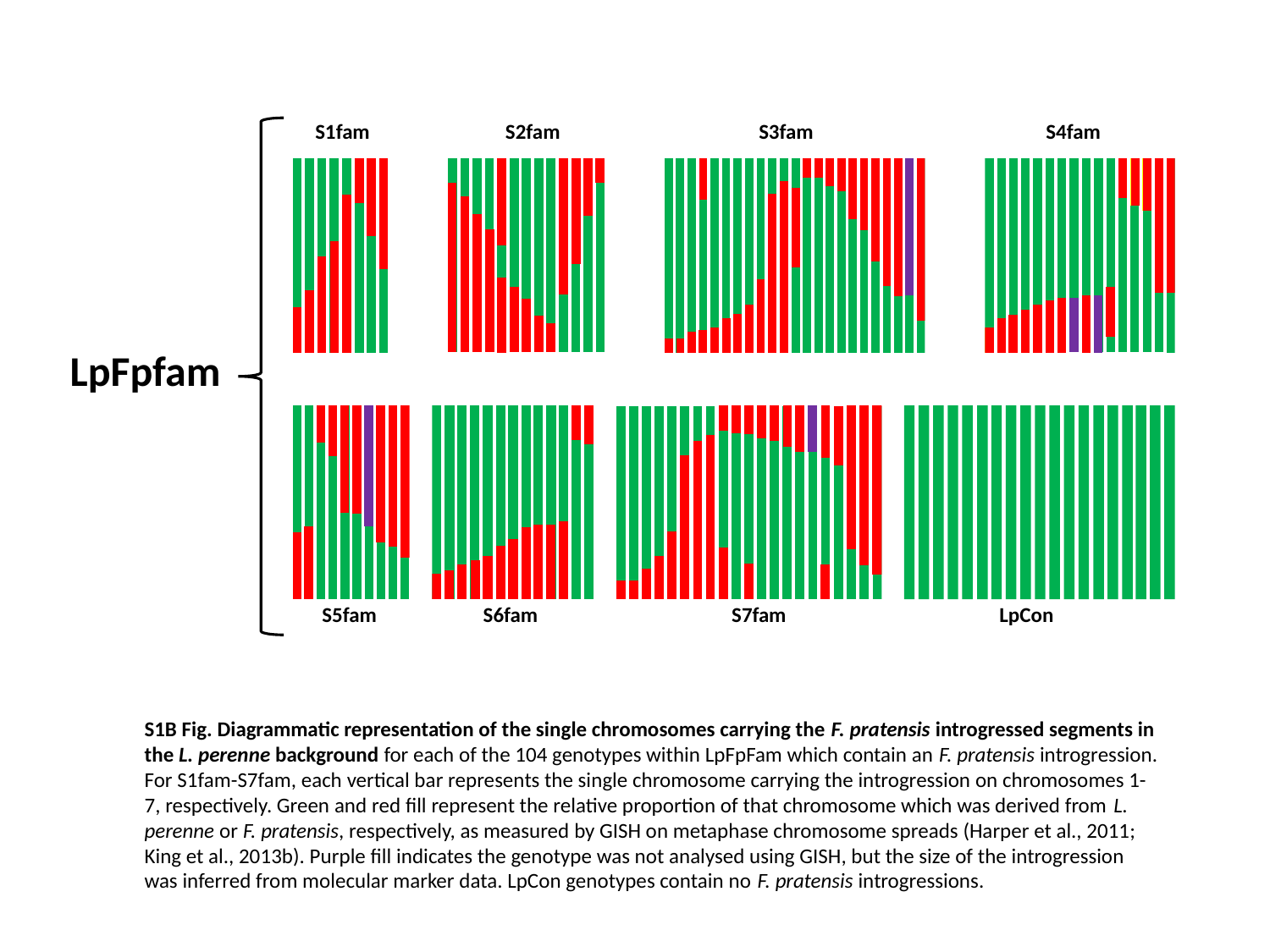

S1fam S2fam S3fam S4fam
 S5fam S6fam S7fam LpCon
LpFpfam
S1B Fig. Diagrammatic representation of the single chromosomes carrying the F. pratensis introgressed segments in the L. perenne background for each of the 104 genotypes within LpFpFam which contain an F. pratensis introgression. For S1fam-S7fam, each vertical bar represents the single chromosome carrying the introgression on chromosomes 1-7, respectively. Green and red fill represent the relative proportion of that chromosome which was derived from L. perenne or F. pratensis, respectively, as measured by GISH on metaphase chromosome spreads (Harper et al., 2011; King et al., 2013b). Purple fill indicates the genotype was not analysed using GISH, but the size of the introgression was inferred from molecular marker data. LpCon genotypes contain no F. pratensis introgressions.
