# Supplementary material for: An investigation of genotype-phenotype association in a festulolium forage grass population containing genome-spanning Festuca pratensis chromosome segments in a Lolium perenne background
Source: PLoS One. 2018 Nov 14;13(11):e0207412. doi: 10.1371/journal.pone.0207412 (PMC6235365; doi:10.1371/journal.pone.0207412)
Supplement: S2 Fig — (PPTX) [file pone.0207412.s002.pptx]

## Slide 1
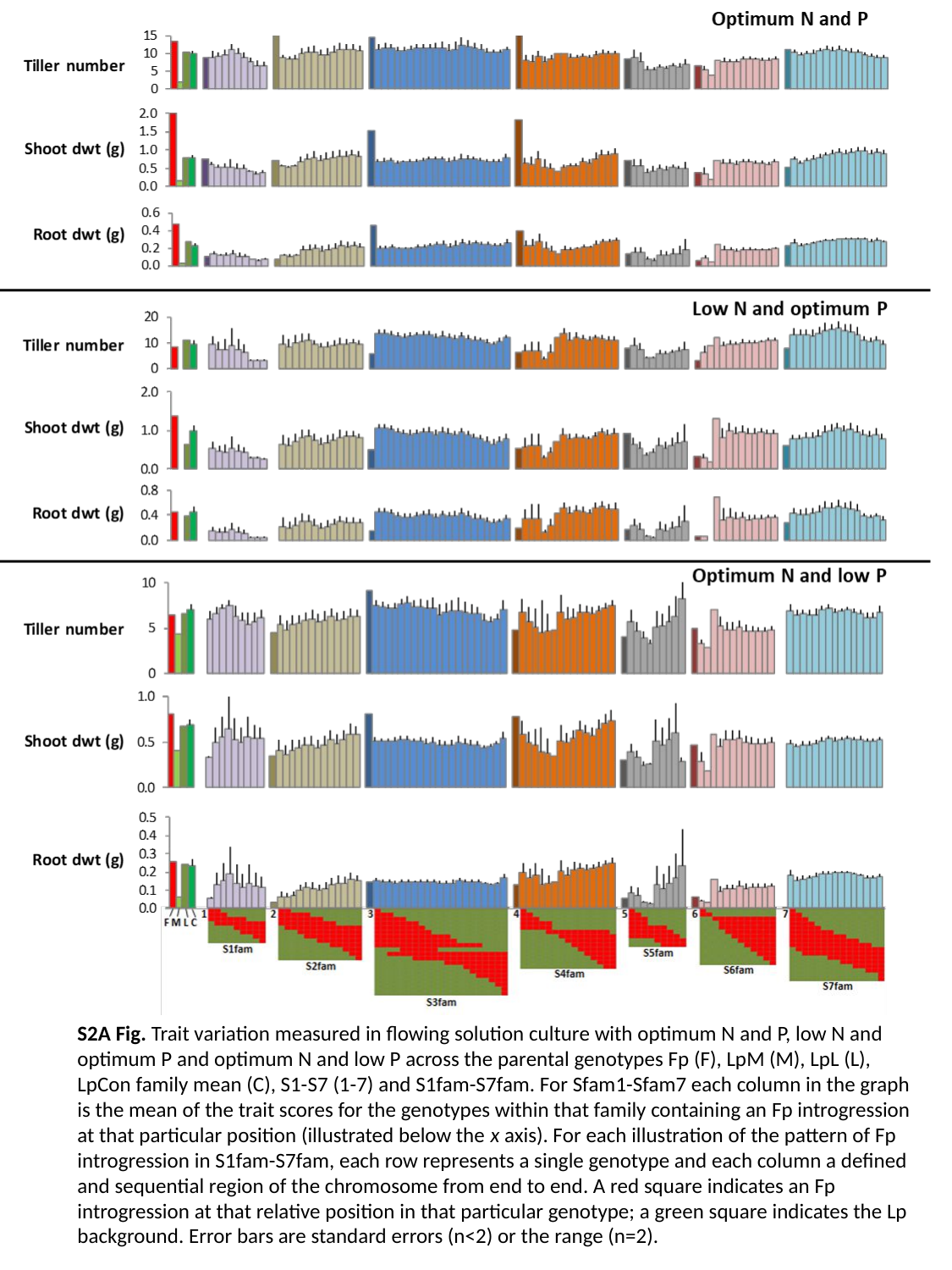

S2A Fig. Trait variation measured in flowing solution culture with optimum N and P, low N and optimum P and optimum N and low P across the parental genotypes Fp (F), LpM (M), LpL (L), LpCon family mean (C), S1-S7 (1-7) and S1fam-S7fam. For Sfam1-Sfam7 each column in the graph is the mean of the trait scores for the genotypes within that family containing an Fp introgression at that particular position (illustrated below the x axis). For each illustration of the pattern of Fp introgression in S1fam-S7fam, each row represents a single genotype and each column a defined and sequential region of the chromosome from end to end. A red square indicates an Fp introgression at that relative position in that particular genotype; a green square indicates the Lp background. Error bars are standard errors (n<2) or the range (n=2).

## Slide 2
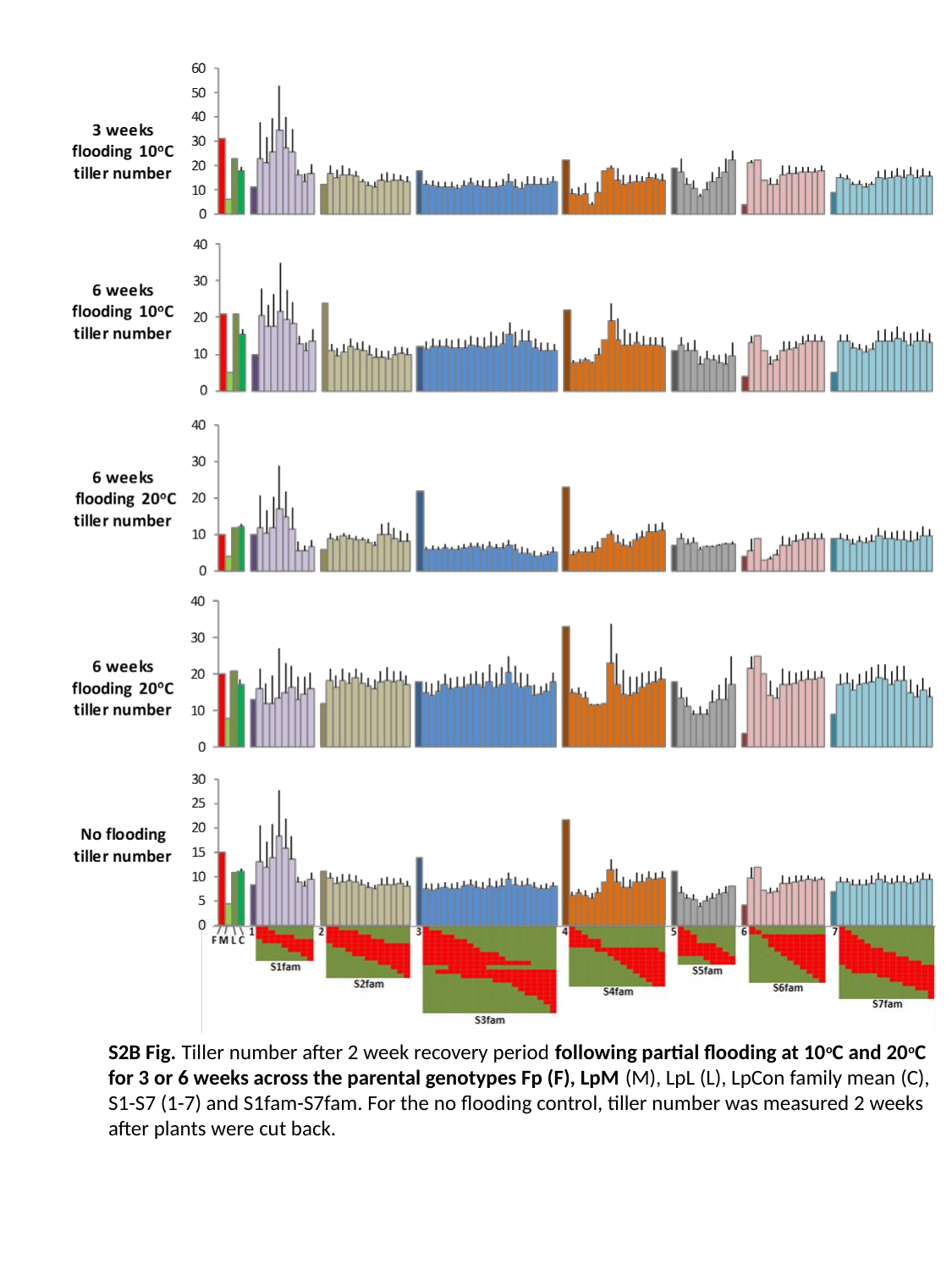

S2B Fig. Tiller number after 2 week recovery period following partial flooding at 10oC and 20oC for 3 or 6 weeks across the parental genotypes Fp (F), LpM (M), LpL (L), LpCon family mean (C), S1-S7 (1-7) and S1fam-S7fam. For the no flooding control, tiller number was measured 2 weeks after plants were cut back.

## Slide 3
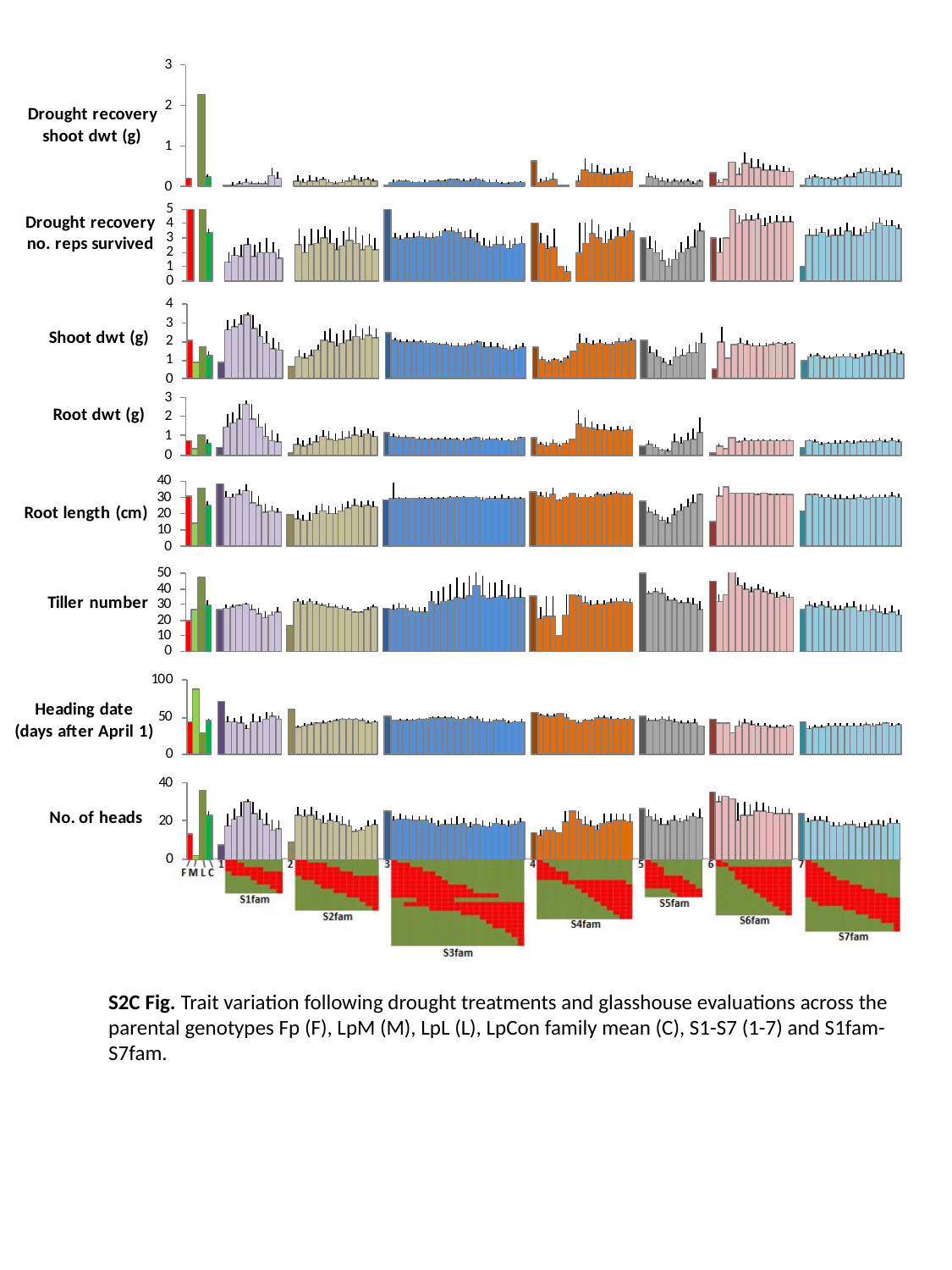

S2C Fig. Trait variation following drought treatments and glasshouse evaluations across the parental genotypes Fp (F), LpM (M), LpL (L), LpCon family mean (C), S1-S7 (1-7) and S1fam-S7fam.
