# Supplementary material for: An investigation of genotype-phenotype association in a festulolium forage grass population containing genome-spanning Festuca pratensis chromosome segments in a Lolium perenne background
Source: PLoS One. 2018 Nov 14;13(11):e0207412. doi: 10.1371/journal.pone.0207412 (PMC6235365; doi:10.1371/journal.pone.0207412)
Supplement: S3 Fig — (PPTX) [file pone.0207412.s003.pptx]

## Slide 1
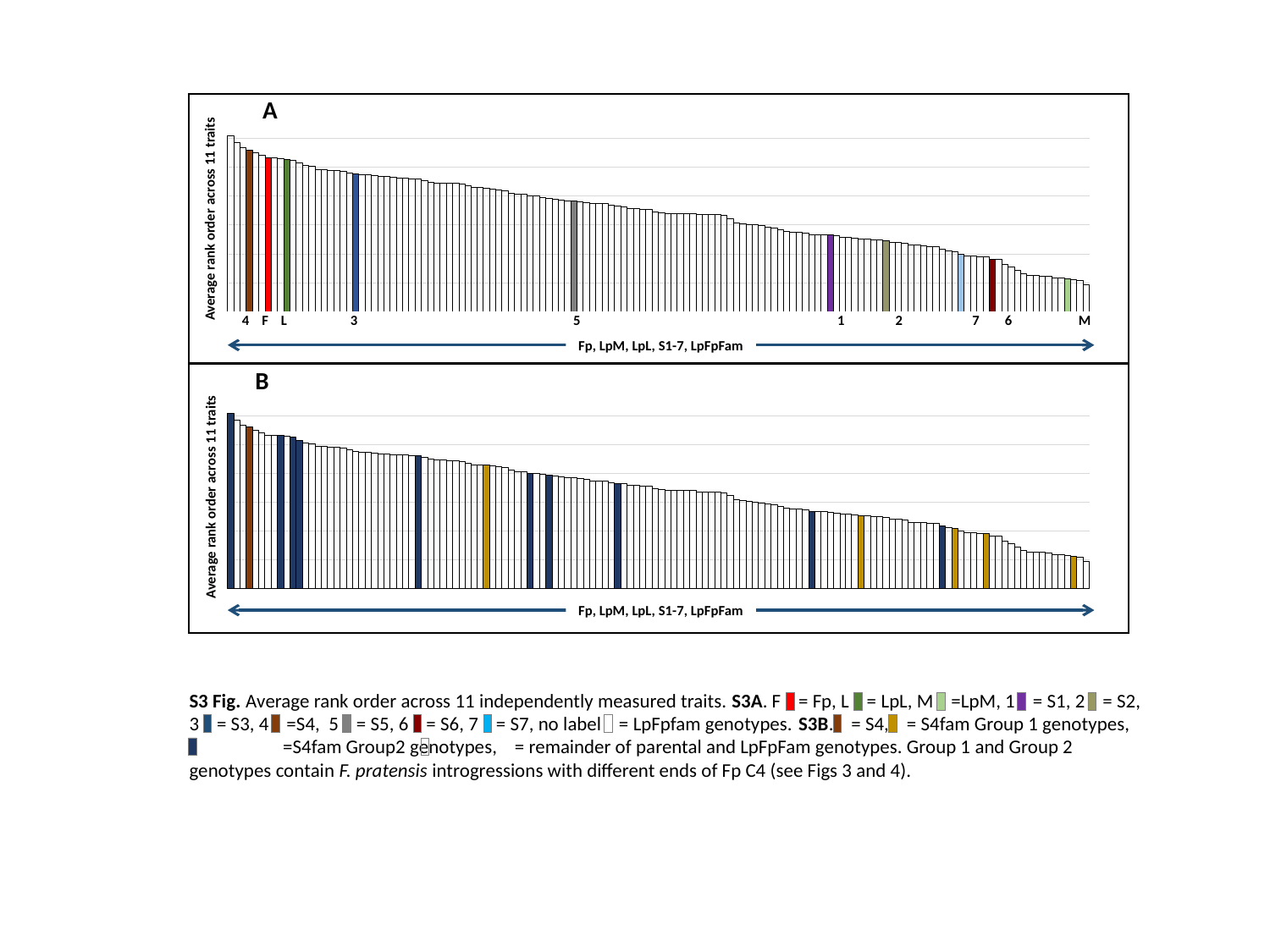

A
Average rank order across 11 traits
4 F L 3 5 1 2 7 6 M
Fp, LpM, LpL, S1-7, LpFpFam
B
Average rank order across 11 traits
Fp, LpM, LpL, S1-7, LpFpFam
S3 Fig. Average rank order across 11 independently measured traits. S3A. F = Fp, L = LpL, M =LpM, 1 = S1, 2 = S2, 3 = S3, 4 =S4, 5 = S5, 6 = S6, 7 = S7, no label = LpFpfam genotypes. S3B. = S4, = S4fam Group 1 genotypes, …=S4fam Group2 genotypes, = remainder of parental and LpFpFam genotypes. Group 1 and Group 2 genotypes contain F. pratensis introgressions with different ends of Fp C4 (see Figs 3 and 4).
