# Supplementary material for: An investigation of genotype-phenotype association in a festulolium forage grass population containing genome-spanning Festuca pratensis chromosome segments in a Lolium perenne background
Source: PLoS One. 2018 Nov 14;13(11):e0207412. doi: 10.1371/journal.pone.0207412 (PMC6235365; doi:10.1371/journal.pone.0207412)
Supplement: S3 Table — (DOCX) [file pone.0207412.s006.docx]

| **S3 Table.** Significant associations between C5 marker Contig50116_879 and indicated traits. | | | | |
| --- | --- | --- | --- | --- |
| **Trials^1^** | **Trait** | **P^3^** | **5% FDR^4^** | **Bf^5^** |
| **FSC NP^2^** | Tiller number | 0.0003 | ns | ns |
|  | Shoot dwt | 0.0009 | ns | ns |
|  | Root dwt | 0.00002 | **YES** | **YES** |
|  |  |  |  |  |
| **FSC nP^2^** | Tiller number | 0.0005 | ns | ns |
|  | Shoot dwt | 0.006 | ns | ns |
|  | Root dwt | 0.0003 | **YES** | ns |
|  |  |  |  |  |
| **FSC Np^2^** | Root dwt | 0.04 | ns | ns |
|  |  |  |  |  |
|  | Shoot dwt | 0.002 | ns | ns |
| **GH^2^** | Root dwt | 0.00006 | **YES** | ns |
|  | Root length | 0.0002 | ns | ns |
|  |  |  |  |  |
|  | Tiller number 3weeks, 10^o^C | 0.0006 | ns | ns |
| **Partial flood** | Tiller number 3weeks, 20^o^C | 0.008 | ns | ns |
|  | Control | 0.02 | ns | ns |
|  |  |  |  |  |
| **DB^2^** | Drought dwt | 0.01 | ns | ns |
|  | Drought_reps_survived | 0.001 | ns | ns |
|  |  |  |  |  |
| ^1^ Trials represent fully independent evaluations.  ^2^Key for environments and treatments: **FSC** = flowing solution culture **NP**, **nP** and **Np** = optimum N and P, reduced N and reduced P, respectively; **GH** = glasshouse; **DB** = drought bins  ^3^P-value from ANOVA or KW test.  ^4^Within 5% Benjamini-Hochberg False Discovery Rate  ^5^Significant after Bonferroni correction | | | | |
